# Cultivation of Mushrooms and Their Lignocellulolytic Enzyme Production Through the Utilization of Agro-Industrial Waste

**DOI:** 10.3390/molecules25122811

**Published:** 2020-06-18

**Authors:** Jaturong Kumla, Nakarin Suwannarach, Kanaporn Sujarit, Watsana Penkhrue, Pattana Kakumyan, Kritsana Jatuwong, Santhiti Vadthanarat, Saisamorn Lumyong

**Affiliations:** 1 Research Center of Microbial Diversity and Sustainable Utilization, Chiang Mai University, Chiang Mai 50200, Thailand; jaturong_yai@hotmail.com (J.K.); suwan.462@gmail.com (N.S.); aew.kritsana@gmail.com (K.J.); santhiti.v@gmail.com (S.V.); 2 Department of Biology, Faculty of Science, Chiang Mai University, Chiang Mai 50200, Thailand; 3 Division of Biology, Faculty of Science and Technology, Rajamangala University of Technology Thanyaburi, Thanyaburi, Pathumthani 12110, Thailand; kanaporn_s@rmutt.ac.th; 4 School of Preclinic, Institute of Science, Suranaree University of Technology, Nakhon Ratchasima 30000, Thailand; watsanapenkhrue@gmail.com; 5 Center of Excellence in Microbial Technology for Agricultural Industry, Suranaree University of Technology, Nakhon Ratchasima 30000, Thailand; 6 School of Science, Mae Fah Luang University, Chiang Rai 57100, Thailand; pattana.kak@mfu.ac.th; 7 Academy of Science, The Royal Society of Thailand, Bangkok 10300, Thailand

**Keywords:** lignocellulosic materials, lignocellulolytic enzymes, mushroom cultivation, solid state fermentation

## Abstract

A large amount of agro-industrial waste is produced worldwide in various agricultural sectors and by different food industries. The disposal and burning of this waste have created major global environmental problems. Agro-industrial waste mainly consists of cellulose, hemicellulose and lignin, all of which are collectively defined as lignocellulosic materials. This waste can serve as a suitable substrate in the solid-state fermentation process involving mushrooms. Mushrooms degrade lignocellulosic substrates through lignocellulosic enzyme production and utilize the degraded products to produce their fruiting bodies. Therefore, mushroom cultivation can be considered a prominent biotechnological process for the reduction and valorization of agro-industrial waste. Such waste is generated as a result of the eco-friendly conversion of low-value by-products into new resources that can be used to produce value-added products. Here, we have produced a brief review of the current findings through an overview of recently published literature. This overview has focused on the use of agro-industrial waste as a growth substrate for mushroom cultivation and lignocellulolytic enzyme production.

## 1. Introduction

The rapidly growing global population and expansion in the agriculture sector and food industries have resulted in the generation of a large amount of agro-industrial waste annually. Agro-industrial waste is defined as the waste that is generated during the industrial processing of agricultural or animal products or the waste obtained from agricultural activities [1,2]. The waste can further be divided into two types, agricultural residues and industrial residues, respectively [2,3,4,5]. Agricultural residues consist of field residues and process residues. Field residues are generated during the crop harvesting process and are made up of leaves, roots, stalks, straw, seed pods and stems. Process residues are generated during the further processing of the crops and are made up of husks, peels, pulp and shells. Asia is the largest producer of agricultural residues at 47%, followed by the United States (29%), Europe (16%), Africa (6%) and Oceania (2%) [6]. Industrial residues are residues that are produced by the food, fruit and vegetable processing industries and include bran, peels, pomace and bagasse. Generally, most agro-industrial waste is disposed of in landfills or burned, leading to various environmental problems and pose potential harm to the health of humans and wildlife [5,7,8]. However, agro-industrial waste can potentially be converted into different high-value products, including biofuels, value-added fine chemicals and cheap energy sources for microbial fermentation and enzyme production [7,8,9]. These waste products can represent a source of energy, as well as sources of carbon. Additionally, this form of waste is a source of the nutrients that are required for mushroom growth and lignocellulolytic enzyme production via solid state fermentation [9,10,11]. Therefore, in this study, we have summarized the current findings on the use of agro-industrial waste as growth substrates for mushroom cultivation and lignocellulolytic enzyme production.

## 2. The Composition of Agro-Industrial Wastes

**Figure 1:**
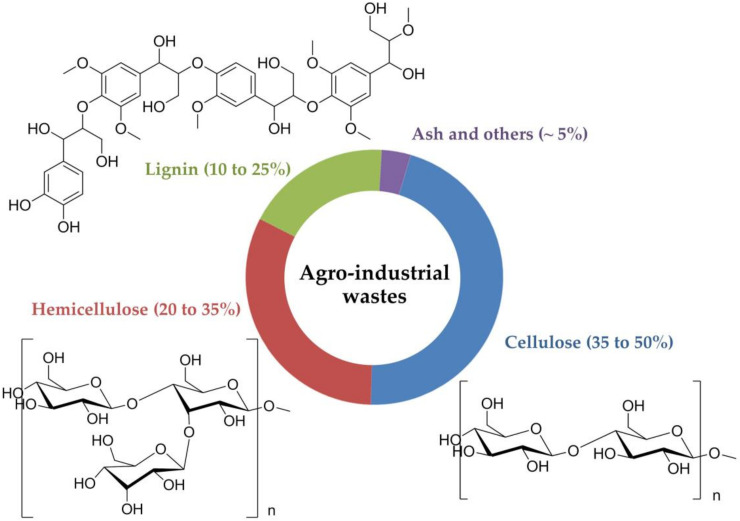
Main composition of agro-industrial wastes.

Agro-industrial waste is a major lignocellulosic component. This form of waste includes cellulose, hemicelluloses and lignin, which are normally referred to as “lignocellulosic materials”. Generally, cellulose is the most abundant component, followed by hemicellulose and lignin (Figure 1).

Cellulose is a homopolymer consisting of a linear chain of several hundred to many thousands of β-anhydroglucose units (β-1,4 linked d-glucose units). Each of the β-anhydroglucose units consists of three hydroxyl groups (OH), one primary (C6 position) and two secondary (C2 and C3 positions) hydroxyl groups, each of which exhibits different polarities and is capable of being involved in the intra- and intermolecular hydrogen bonds [12,13]. The intra- and inter-chain hydrogen bonding network makes cellulose a relatively stable polymer and gives the cellulose fibrils high axial stiffness [14].

Hemicellulose is a heteropolymer consisting of a polysaccharide backbone. Its structure greatly varies depending on the sugar units, chain length and the branching of the chain molecules. Typical binding sugars in hemicelluloses are pentoses (xylose and arabinose), hexoses (mannose, glucose, and galactose), hexuronic acids (4-*O*-methyl-d-glucuronic acid, galacturonic acid, and glucuronic acid), small amounts of rhamnose and fucose, and an acetyl group [12]. These binding sugars can assemble into a range of various hemicellulose polysaccharides, such as galactan mannans, xylans, xyloglucan and β-1,3/1,4-glucans [12,15].

Lignin is a rigid aromatic, amorphous and hydrophobic polymer that has been recognized as a highly branched polymer with a variety of functional groups, such as aliphatic, phenolic hydroxyls, carboxylic, carbonyl, and methoxyl groups. These functional groups give lignin a unique and very complex structure [16,17,18]. The nature of the lignin polymerization reactions results in the formation of a three dimensional, highly-branched, interlocking network of essentially infinite molecular weight. Lignin composition and content are influenced by plant species and the environment [17,18].

The composition of cellulose, hemicellulose and lignin in agro-industrial waste depends upon the species, tissue and maturity of the plant [2,4,5,12]. The values of the main components in some agro-industrial waste are shown in Table 1.

## 3. Mushroom Cultivation on Agro-Industrial Wastes

**Figure 2:**
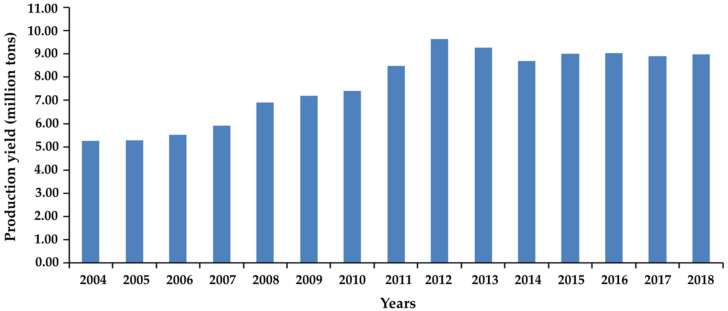
Data of global mushroom production during 2004–2018 from FAOSTAT [56].

Mushroom cultivation is widespread throughout the world and its global production has significantly increased since 2010 (Figure 2). The Food and Agriculture Organization Statistical Database (FAOSTAT) reported that China is the largest mushroom producer, followed by the United States of America and the Netherlands, with global production in 2018 reaching almost 8.99 million tons. The trend to increase mushroom production is expected to continue in the future. 

**Figure 3:**
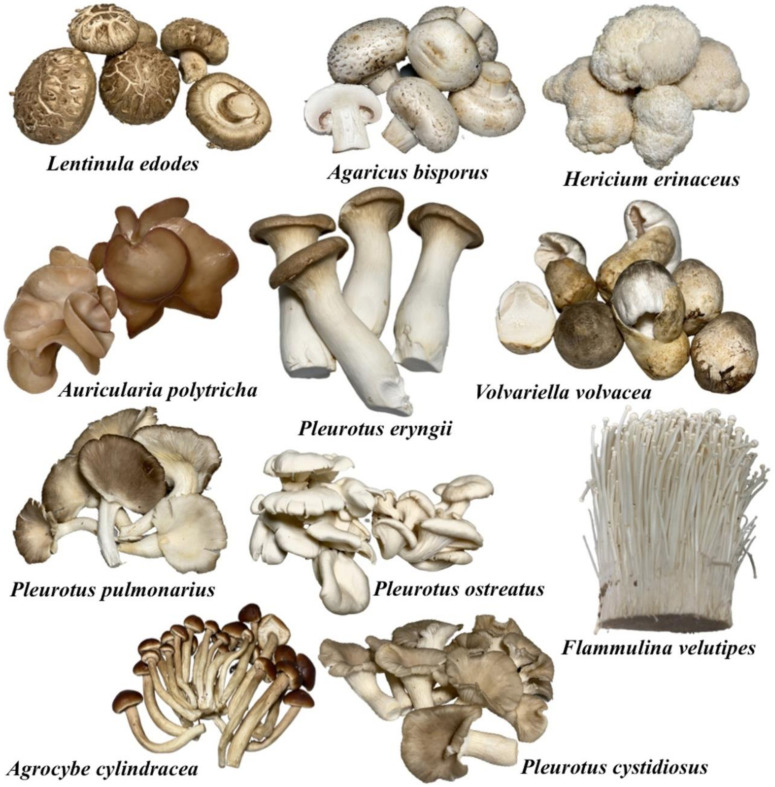
Examples of some commercially important cultivated mushrooms.

Edible mushrooms are also considered a healthy food because they are rich in proteins, carbohydrates, fiber, vitamins and minerals while being low in fat [57,58]. Normally, the range of protein, carbohydrate and fat contents in mushrooms is 15–35%, 35–70% and less than 5%, respectively [58]. Notably, several species of edible mushrooms are important because of their medicinal properties. Some edible mushrooms appear to be active against human pathogens, cancer, diabetes, hypertension, hypercholesterolemia conditions and tumors [57,58,59]. Today, more than 50 species of edible mushrooms have been commercially cultivated throughout the world. Most commercial edible mushrooms belong to the genera *Agaricus*, *Agrocybe*, *Auricularia*, *Flammulina*, *Ganoderma*, *Hericium*, *Lentinula*, *Lentinus*, *Pleurotus*, *Tremella*, and *Volvariella* (Figure 3). The top four globally cultivated edible mushrooms include the genera *Lentinula* (shiitake and relatives), *Pleurotus* (oyster mushroom), *Auricularia* (wood ear mushroom) and *Agaricus* (button mushroom and relatives) [54,60]. In 2017, world mushroom production was divided among several genera: *Lentinula* (22%), *Pleurotus* (19%), *Auricularia* (18%), *Agaricus* (15%), *Flammulina* (11%), *Volvariella* (5%) and others (10%) [60]. Most of the cultivated edible mushrooms are saprophytic fungi (decomposers) and able to degrade lignocellulosic materials by producing extensive enzymes (especially lignocellulolytic enzymes). They are then able to use these materials as nutrients for their growth. Thus, mushroom cultivation is often associated with the recycling of vast amounts of agro-industrial waste [2,3,4,54].

Agro-industrial wastes (both agricultural residue and industrial residue) have been used as substrates in mushroom cultivation. Most agro-industrial waste is defined as low nitrogen content materials. The carbon/nitrogen (C/N) ratio in agro-industrial waste is varied among different types (Table 1), and it is an important factor in mushroom cultivation. This ratio has a critical influence on mycelium growth, mushroom weight, yields and protein content in the fruiting body of mushrooms [11,61,62]. Therefore, low-level nitrogen substrates for mushroom cultivation are necessary in that they add organic (cereal bran, cereal shell, soybean meal and manure) or inorganic (ammonium chloride and urea) nitrogen supplements [63,64]. Several previous studies have found that the protein content in the fruiting body of mushrooms depends upon both the chemical composition and the C/N ratio of substrates, as well as the species of mushroom being cultivated [1,64,65,66]. Different mushroom species require different C/N ratios in the cultivation substrate in order to obtain the highest production yield, as is shown in Table 2. Moreover, the addition of various supplements, e.g., epsom salts (MgSO_4_∙7H_2_O), gypsum (CaSO_4_·2H_2_O) and limestone (calcium carbonate, CaCO_3_), in the substrates also support the mycelia growth and fruiting body production of mushrooms [11,61,67].

Biological efficiency (BE), which is used to evaluate the efficiency of substrate conversion in mushroom cultivation, is calculated as the percentage ratio of the fresh weight of harvested mushrooms over the dry weight of the cultivation substrate [67]. A high BE value ensures a high possibility of utilizing substrates for mushroom cultivation [67,82]. In considering the profitability of mushroom cultivation, the BE value must be over 50%. Utilization of agro-industrial waste for the cultivation of mushrooms has resulted in the production of edible proteins for human consumption [2,7,11]. Cultivation methods for edible mushrooms vary considerably around the world and a variation in the chemical composition of a particular cultivated mushroom has been observed in various studies. This may be related to the specific mushroom species, the growing substrate and the relevant environmental conditions [1,2,11]. Many studies have been conducted to test the ability of mushrooms to grow on different agro-industrial forms of waste, such as wheat straw, barley straw, oat straw, rice straw, corn straw, corn cob, banana leaves, sawdust, sugarcane bagasse, soya stalk and sunflower stalk. A combination of agro-industrial waste can be used in mushroom cultivation. The main results regarding the cultivation of edible mushrooms on different agro-industrial waste, and their proximate composition values, are shown in Table 3 and Table 4.

## 4. Lignocellulolytic Enzyme Production by Mushroom Using Agro-Industrial Wastes

**Figure 4:**
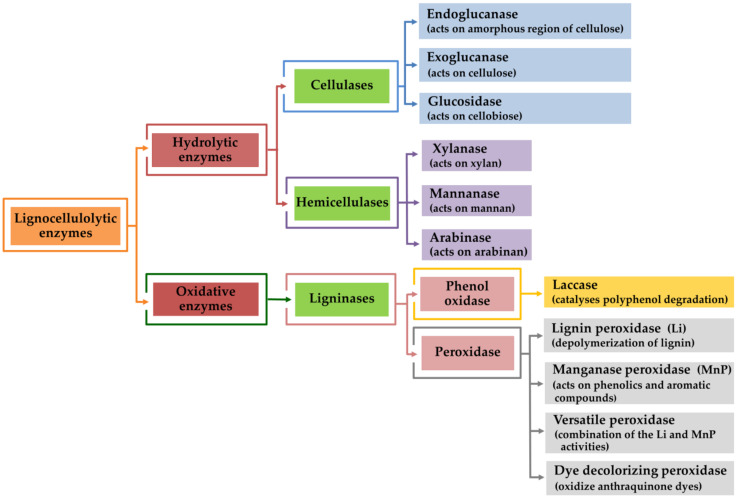
Scheme of the main enzymes involved in the lignocellulosic degradation process.

The decomposition of lignocellulosic materials is carried out by decomposers such as bacteria, microfungi, mushrooms, earthworms, and woodlice, all of which play an important role in the terrestrial carbon cycle [130,131,132]. Lignocellulose is a composite of three main biopolymers: cellulose, hemicellulose and lignin. Due to the different bonding functions that exist among these polymers, lignocellulose degradation requires the synergistic action of multiple carbohydrate-active enzymes. These are involved in the assembly and breakdown of glycosidic bonds [132,133,134]. The degradation of lignocellulosic biomass is achieved through cooperative activities of hydrolytic and oxidative enzymes [134,135,136], as is shown in Figure 4. The hydrolytic system is responsible for cellulose and hemicellulose degradations, whereas the oxidative system is known to participate in lignin degradation.

### 4.1. Cellulose Degradation Enzymes

**Figure 5:**
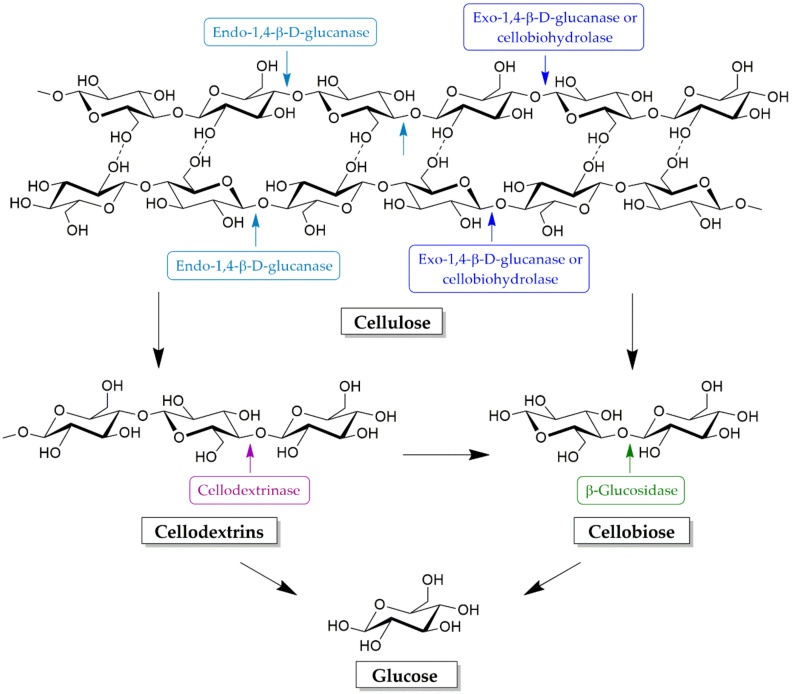
Enzymes involved in cellulose degradation.

Commonly, cellulose hydrolysis requires a combination of three main types of cellulase: endo-1,4-β-d-glucanase (endoglucanase, EC 3.2.1.4), exo-1,4-β-d-glucanase or cellobiohydrolases (exoglucanase, EC 3.2.1.91) and β-glucosidase (β-d-glucoside glucanhydrolase, EC 3.2.1.21), in order to convert cellulose into oligosaccharides, cellobiose, and glucose [137,138]. The degradation of cellulose by various cellulase enzymes is diagrammed in Figure 5. Endoglucanases preferentially hydrolyze internal β-1,4-glucosidic linkages in the cellulose chains, generating a number of reducing ends [138,139]. This enzyme also acts on cellodextrins, which are the intermediate product of cellulose hydrolysis, and converts them to cellobiose and glucose. Exoglucanases release cellobiose from the reducing end or the nonreducing end of the cellulose chain, facilitating the production of mostly cellobiose which can readily be converted to glucose by β-glucosidases [136,140,141]. These enzymes may also act on cellodextrins and larger cello-oligosaccharides, in which case they are commonly named cellodextrinases [142]. Oligosaccharides released as a result of these activities are converted to glucose by the action of cellodextrinases (EC 3.2.1.74), whereas the cellobiose released mainly by the action of cellobiohydrolases is converted to glucose by β-glucosidases [139].

Cellulases are produced in a wide range of organisms such as plants, some animals, and certain microorganisms including protozoans, bacteria, and fungi. Among these organisms, fungi have been studied extensively for their cellulase producing capabilities, such as the genera *Aspergillus*, *Penicillium*, *Rhizopus* and *Trichoderma* [143,144,145,146]. However, mushrooms are the most potent degraders of natural lignocellulosic waste. They are mostly grown on litter, dead wood, or in soil and nature-rich cellulose [9]. Several previous reports have found that various mushrooms species can produce cellulase via solid state fermentation (SSF) of agricultural or natural lignocellulosic waste [147,148,149]. Many agricultural or natural lignocellulosic solid waste, especially different kinds of straw (wheat, sorghum, rice) and sawdust (oak and pine), were used as a substrate or source for mushroom growth and cellulases production [150,151,152,153]. Furthermore, other forms of lignocellulosic waste, such as peanut hulls, mandarin peels, cotton waste, corn stovers and tree leaves (*Fagus sylvatica*), have also been used as substrates to determine cellulase activity [150,154,155]. The high-value potential of these forms of waste is encouraging as they can be sources that support the growth and cellulases production of different mushroom species, namely *Ganoderma*, *Grifola*, *Lentinula, Lentinus*, *Pleurotus*, *Piptoporus* and *Trametes* by SSF [152,156,157,158,159]. Different agricultural or natural lignocellulosic forms of waste that have been fermented by various mushroom species are summarized in Table 5.

Cellulase activity is mainly tested using a reducing sugar assay to determine cellulase hydrolysis activity at the end of the production process [169]. The common enzyme activity assays consist of total cellulase assays, endoglucanase assays, exoglucanase assays and β-glucosidase assays [140]. Filter paper assay (FPA) is widely used to determine total cellulase activity. The degree of filter paper activity is determined as the micromole of glucose equivalent liberated per minute of culture filtrate under assay conditions [170]. Endoglucanase activity can be measured using the carboxymethyl cellulose (CMC) as a substrate. This carboxymethyl cellulase (CMCase) is mainly measured by examining the reducing sugars of enzymatic reactions with CMC based on the procedure described by [171]. The exoglucanase activity mainly uses commercial Avicel as a substrate for measuring the activity [169]. The β-glucosidase assay can be measured based on the procedure of Kubicek [172] using chromogenic and nonchromogenic substrates such as *p*-nitrophenol-β-glucoside (pNPG) and cellobiose, respectively [173,174]. Moreover, various reducing sugar assays, for instance, 3,5-dinitrosalicylic acid (DNS), glucose oxidase (GOD) and high-performance liquid chromatography were also used.

### 4.2. Hemicellulose Degradation Enzymes

Hemicelluloses are usually classified based on the backbone sugars present in the structural polymer with typical glucose galactose, xylose, mannose, and arabinose. The principal hemicelluloses are comprised of xyloglucans, xylans, mannans, glucomannans, and mixed linkage β-glucans [175,176]. In order to digest hemicellulose, microorganisms need to be able to produce a variety of enzymes to hydrolyze complex substrates with a synergistic action. Hemicellulolytic enzymes or hemicellulases are glycoside hydrolases or carbohydrate esterases that are responsible for polysaccharide degradation. The enzymes include xylanase (EC 3.2.1.8), β-xylosidase (EC 3.2.1.37), α-arabinofuranosidase (EC 3.2.1.55) α-glucuronidase (EC 3.2.1.139), and β-mannosidases (EC 3.2.1.25) [134,138].

#### 4.2.1. Xylanases

**Figure 6:**
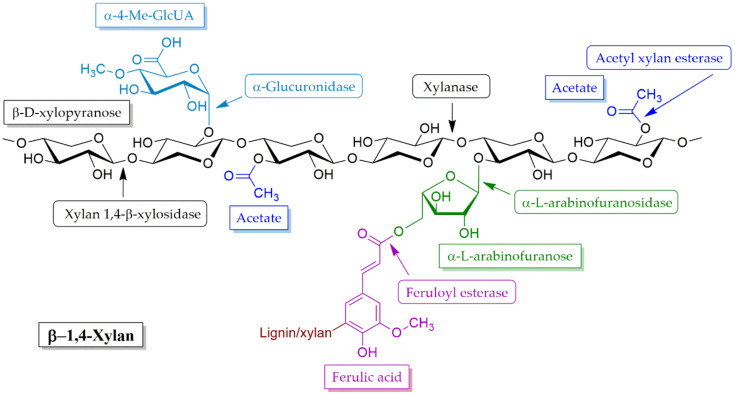
Enzymes involved in xylan degradation.

Xylan is a heteropolysaccharide and a major hemicellulose. The main chain of xylan consists of β-1,4-linked d-xylopyranosyl residues, which are partially replaced with *O*-acetyl, l-arabinosyl and 4-*O*-methyl-d-glucuronic acid. The xylan backbone is substituted by different side chains with l-arabinose, d-galactose, d-mannoses, and glucouronic acid linked by glysosidic bonds and ester bonds with ferulic acid [177,178,179,180]. Biodegradation of xylan requires diverse modes of action of hydrolytic enzymes. Xylanases are a group of glycoside hydrolase enzymes that breakdown hemicelluloses through the degradation of the linear polysaccharide xylan into xylose by catalyzing the hydrolysis of the glycosidic linkage (β-1,4) of xylosides. The xylanolytic enzyme system includes a mixture of endo-1,4-β-xylanases also called endo-xylanases, β-xylosidases, α-arabino- furanosidases, α-glucuronidases and acetylxylanases, which attach to the specific site of xylan as is displayed in Figure 6 [181,182]. Endo-xylanases randomly hydrolyze β-1,4-xylanopyranosyl linkages of xylan to form xylo-oligosaccharides, xylotriose, xylobiose and xylose. The hydrolysis of xylans is not attacked randomly but depends upon the degree of branching, chain length, and presence of substituents in the substrate molecule [183].

β-Xylosidase attacks from the non-reducing end of xylo-oligosaccharides, xylotriose or xylobiose, that are generated by the action of endo-xylanase and ultimately liberate xylose sugar (Figure 6). Biomass can be used as a substrate for this enzyme production process. However, a limitation of the commercial application of this substance is related to various factors such as their physical limitations, the limited hydrolysis of xylans due to their diverged branched nature, the fact that their enzymes are associated with a narrow pH range and thermal instability, their end product inhibition levels and the cost of enzyme production. These comprise the unreachability of substrates to xylanase enzymes [178]. The use of substrates with agricultural or industrial biomass for enzyme production serves as an alternative way to overcome the limitations of the costs of enzyme production; however, biomass pretreatment is sometimes needed to improve efficiency in the practical hydrolysis of biomass.

Many microorganisms, such as fungi, bacteria and yeast, can degrade hemicellulose by producing xylanases. A determination of xylanase activities can be analyzed by several methods. The plate assay has been used for decades as a primary screening method to select xylanase producing strains. The screening strains are cultured on agar medium containing xylan as their carbon source until clear zones are observed (the xylan hydrolysis area) after being stained with Congo red dye [183] or Gram’s iodine solution [184]. Plate assay methods rely on interactions between a dye and a polymeric substrate for the indirect detection of hydrolysis but require the use of relevant controls and independent confirmation of the relevant enzymatic activities. Xylans, such as oat spelt, beech wood [185], and birch-wood xylans [186,187], was used as a substrate to determine endo-xylanase activity. The enzyme activities were determined from the presence of reducing sugars as xylose equivalents liberated from the enzymatic hydrolysis by the DNS method [188] or the Nelson [189] and Somogyi [190] methods. However, xylans obtained from natural sources contain not only xylose residues but also arabinose and glucuronic acid residues. Thus, comparisons of xylanase activity in various studies have been difficult. Xylanase activity varies according to the source of the xylans. Other types of substrates can be applied. Specifically, *p*-nitrophenyl-glycoside substrate (*p*-nitrophenyl esters with substrate) can be used as a chromogenic substrate for the calorimetric assay of β-xylosidase activity. The substrate is colorless in neutral or alkaline solution. After enzymatic hydrolysis, *p*-nitrophenol is liberated as alkaline pH develops a yellow color that is suitable for the quantitative measurement of the enzyme activity. 

Multifunctional xylanolytic enzyme system is relatively common in fungi, actinomycetes and bacteria [190,191]. A large variety of industrial xylanase enzymes are produced from various kind of microorganisms [192]. SSF with batch processing has been used for the utilization of agro-industrial waste [193]. However, very few studies have reported on the xylanolytic enzymes obtained from mushroom on SSF (Table 6). These potential outcomes provide opportunities for scientists to explore the hydrolytic potential of xylanase for the efficient saccharifcation of lignocellulosic biomass from mushroom cultivation.

#### 4.2.2. Mananases

The two most important and representative hemicelluloses are xylans and mannans. Mannans are polysaccharides that consist of mannose-based backbones linked by β-1,4-linkage with variable degrees of side substitutions. These polysaccharides are renewable resources and their enzymatic conversion is of great interest in the field of lignocellulose biotechnology [194]. The enzyme breakdown of mannans is accomplished with β-mannanase (β-1,4-d-mannan mannohydrolase, EC 3.2.1.78) as it randomly attacks the internal β-1,4-d-mannopyranosyl linkage within the main chain of various mannan-based polysaccharides, such as galactomannans, glucomannans, and galactoglucomannans, to release mannooligosaccharides (MOS), manotetrose, manotriose and manobiose [176].

**Figure 7:**
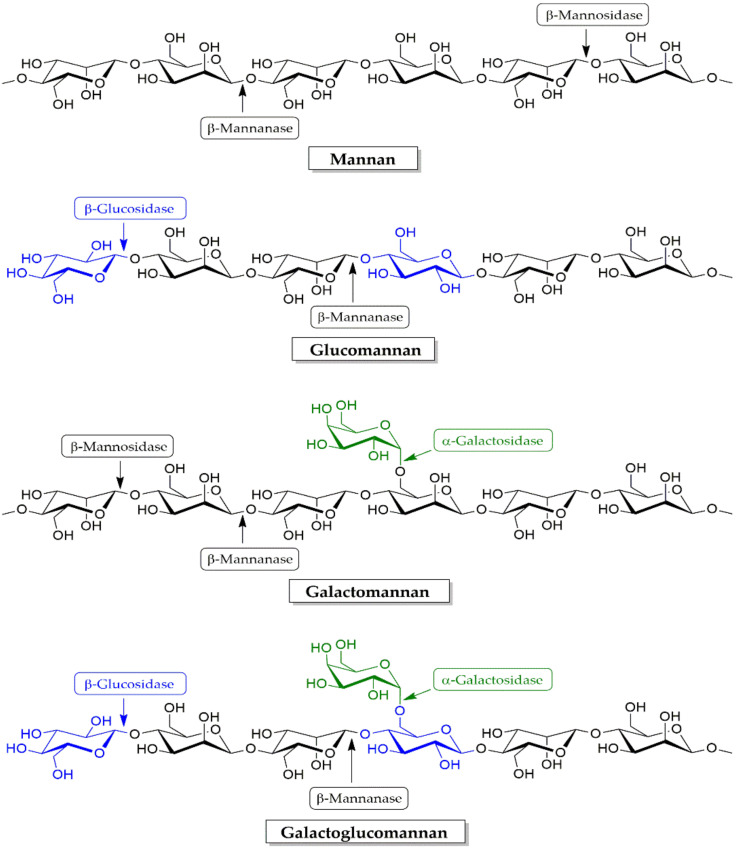
Enzymes involved in mannan degradation.

The degradation of the mannan backbone is performed by the action of β-mannanases, and the further degradation requires β-mannosidase (β-1,4-d-mannopyranoside hydrolase, EC 3.2.1.25) to hydrolyze the terminal ends (non-reducing ends) of MOS into sugar-based mannose. Subsequently, β-glucosidases remove 1,4-glucopyranose units at the non-reducing ends of the oligomers derived from the degradation of glucomannan and galactoglucomannan [171,205] as is shown in Figure 7. Xylanases and mannanases are important enzymes for the hydrolysis of hemicelluloses. β-mannan is found in many feedstuffs including soybean meal, palm kernel meal, copra meal, and sesame meal and other leguminous feeds [206]. β-Mannanases are widely applied to randomly hydrolyze the β-1,4-mannopyranoside linkage of mannan-based polysaccharides in many industries.

#### 4.2.3. Arabinanases

**Figure 8:**
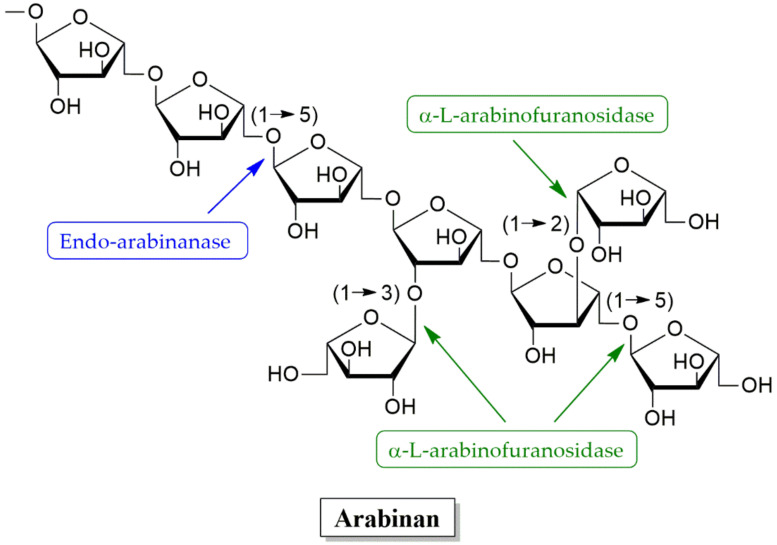
Enzymes involved in arabinan degradation.

Arabinanases are a group of hydrolytic enzymes that include endo-arabinanases (EC 3.2.1.99), arabinosidases (EC 3.2.1.55), and α-L-arabinofuranosidase. These work synergistically to generate l-arabinose from arabinan as is shown in Figure 8 [207,208,209,210,211]. The biodegradation of xylan requires the cooperation of xylanases, β-xylosidase, α-l-arabinofuranosidase, α-glucuronidase, and acetylxylanases [181,182]. The removal of the side groups of xylans is catalyzed by α-l-arabinofuranosidases (E.C. 3.2.1.55), α-d-glucuronidases and acetylxylan esterases, which remove acetyl and phenolic side branches and act synergistically on the complex polymer [178]. Fungi produce extracellular arabinanases, a group of hydrolytic enzymes that include α-l-arabinofuranosidases and endo-arabinanases to specifically release l-arabinose from polysaccharides including xylans and pectin [212]. Importantly, α-l-arabinofuranosidases catalyze the hydrolysis of α-l-arabinofuranosidic linkage at terminal non-reducing- α-l-1,2-, α-l-1,3- and α-l-1,5-arabinofuranosyl residues obtained from different oligosaccharides and polysaccharides (α-l-arabinosides, arabinans, arabinoxylans, and arabinogalactans) and act synergistically with other hemicellulases to completely breakdown hemicellulose [212,213]. The l-arabinofuranoside substitutions on xylan strongly inhibit the action of xylan-degrading enzymes, thus preventing the complete degradation of xylan to xylose units [213]. The α-l-arabinofuranosidases can be found in plants, bacteria and fungi [186].

The colorimetric method is used to determine α-l-arabinofuranosidases activity. Notably, the *p*-nitrophenol-linked substrate, 4-nitrophenyl α-l-arabinofuranoside, is used for the enzyme assay by determining the amount of *p*-nitrophenol released from the enzyme-substrate reaction [186,214,215]. Arabinoxylans, such as wheat four arabinoxylan and sugar beet arabinan, is also used for the determination of enzyme activity [180] by monitoring the generation of arabinose from polysaccharide substrates. Liberated arabinose can be determined by the DNS method [187].

### 4.3. Lignin Degradation Enzymes

Lignin degradation is the primordial step in lignocellulose degradation enabling the accessibility of cellulose and hemicellulose [216,217]. Ligninolytic microorganisms can degrade lignins via the secretion of oxidative enzymes, such as peroxidases and laccases, or by producing a source of heterogeneous aromatics. Ligninolytic enzymes or ligninases are mainly comprised of laccases (Lac, EC 1.10.3.2), lignin peroxidases (LiPs, EC 1.11.1.14), manganese peroxidases (MnPs, EC 1.11.1.13), versatile peroxidases (VPs) and dye decolorizing peroxidases (DyPs, EC 1.11.1.19) [116,218]. These enzymes display less substrate specificity than cellulases and hemicellulases [124,218,219]. Additionally, Lac, LiP and MnP, and many other enzymes, such as aromatic acid reductase, aryl alcohol dehydrogenase, catalase aromatic aldehyde oxidase, dioxygenase, quinone oxidoreductase, vanillate hydroxylase, veratryl alcohol oxidase and versatile peroxidase, are also involved in lignin digestion [219].

Mushroom species are most frequently reported as Lac and MnP producers and least frequently reported as LiP and VP producers. Previous publications have reported that *T. versicolor* [220] and *Bjerkandera adusta* [221] produce both oxidase (Lac) and peroxidase (MnP and LiP). *Lentinula edodes* [222], *P. eryngii* [223] and *Ceripotiopsis subvermispora* [224] are lignin-degrading mushrooms that use Lac and at least one of the peroxidases. Only Lac was produced from *S. commune* and only peroxidases were produced from *Phanerochaete chrysosporium* [225,226]. Several publications have reported that *Ph. chrysosporium* is an excellent lignin decomposer, and it has been suggested for its commercial use. The ligninolytic enzymes were fermented in SSF using different agro-industrial waste, as is shown in Table 7.

#### 4.3.1. Laccases

**Figure 9:**
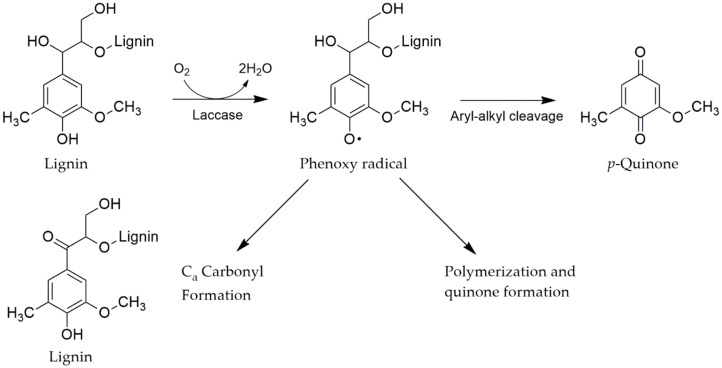
Typical reaction of laccase on phenols oxidation modifled from Minussi et al. [244].

Laccases are a group of multicopper containing enzymes belonging to the blue multicopper oxidase family. The enzymes are also known as polyphenol oxidases, among which laccases oxidize one-electron of phenolic compounds with an associated reduction of oxygen to water as a by-product [240,241]. The enzymes do not require H_2_O_2_ for substrate oxidation. Lac can oxidize both phenolic aromatic compounds such as methylated phenol, aromatic amine and non-phenolic aromatic compounds such as veratryl alcohol in lignin to form phenoxy-free radicals. In this way, lignin degradation and lignin structural conversion can occur [242], as is shown in Figure 9. This oxidation process produces phenoxy radicals that can be converted to quinine by a second enzyme catalyzed reaction [166,243].

Laccases contain four copper ions except for the laccase that is obtained from *Phlebia radiata*, which has only two copper ions [245]. There are three types of Lac depending on the copper number at the active site [246]. Type I: copper does not bind O_2_ but functions only as an electron transfer site. The type I copper center consists of a single copper atom that is coordinated with two histidine residues and one cysteine residue. In some cases, a methionine motif serves as a ligand with a trinuclear center. The Type II copper center has two histidines and a water molecule that serves as a ligand. The type III copper center contains two copper atoms that each possess three histidine ligands and are linked to one another via a hydroxide bridging ligand. Most of the studies on Lac have reported that the fungi and mushrooms present in basidiomycetes, deuteromycetes and ascomycetes act as Lac producers [247]. Among these fungi, the major Lac producers are white-rot fungi in basidiomycetes [246]. White-rot fungi *Pycnoporus cinnabarinus*, *Phlebia radiate*, *P. ostreatus*, and *T. versicolour* are also known to produce one isoform of Lac [248]. Cotton stalks, aromatic compounds, wood, and plant extracts were found to be inducers for Lac production [249]. For Lac production, extracted 3-hydroxyanthranilic acid (3-HAA) obtained from wheat straw was found to be a potential Lac stimulator [250]. The mixture of coffee pulp and urea was also able to enhance the Lac activity in *Py. sanguineus* culture. Some researchers have found a novel Lac obtained from *T. orientalis*, which has a molecular mass of 44.0 kDa. The enzyme contains a typical copper II binding domain and shares three N-glycosylation sites. But it has no copper I binding domain [251] Dias and colleagues [252] have reported a new zymogram dried 2,2’-azino-bis(3-ethylbenzo- thiazoline-6-sulfonic acid) (ABTS)-impregnated discs assay for laccase activity detection, which is associated with easy assay and rapid screening. The laccase activity was determined at a wavelength of 420 nm by measuring the oxidation of ABTS in phosphate citrate buffer at a pH value of 4.0 [253]. The other guaiacol assay has been reported for laccase assay by Kalra et al. [254] to measure the reddish-brown color development at 450 nm as a consequence of the oxidation of guaiacol by Lac.

#### 4.3.2. Lignin Peroxidases

Lignin peroxidase (LiP) belongs to the family of oxidoreductases. LiP has ferric heme as an electron donor which is able to reduce oxygen molecules to hydrogen peroxidase and superoxides. LiP-Fe(III) uses H_2_O_2_ to oxidize aryl cation radicals as the initial substrate. The resulting amount of the lacked electron LiP is not stable and draws electrons from the substrate for stability of the electron condition. Finally, the oxidation cycle ends when LiP-Fe(IV) is turned to the resting ferric state [255]. This reaction exhibits a degree of stoichiometry of one H_2_O_2_ compound consumed per the amount of aldehyde formed. LiP is a strong oxidant and is non-specific with a substrate. It can degrade both structures of phenolic aromatic and non-phenolic aromatic compounds. Veratryl alcohol was found to be an inducer of LiP that was produced from white-rot fungi. The molecular weight of LiP was approximately 41 kDa and contains one mole of Fe protoporphyrin IX. It is a glycoprotein with isoelectric point (pI) as 3.2–4.0 that displays high redox potential activity and an optimum pH value at 3.0 [250]. 

**Figure 10:**
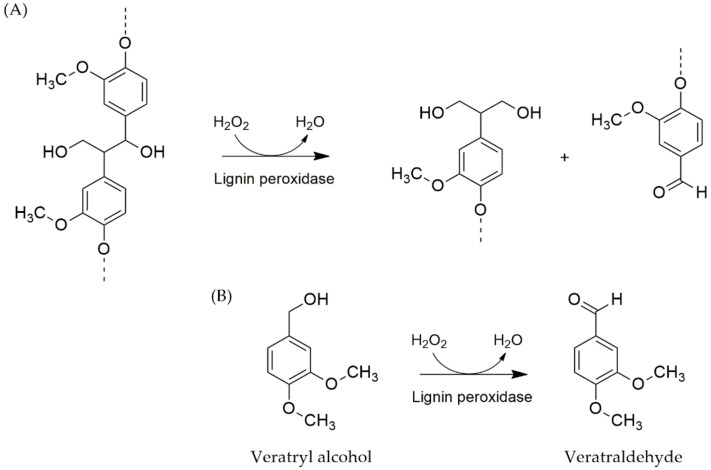
General reaction catalyzed by lignin peroxidase. (**A**) cleavage of C-C of lignin, (**B**) oxidation of veratryl alcohol is generally used to estimate the lignin peroxidase activity.

There are two methods for lignin peroxidase detection [250]. One involves the measurement of veratraldehyde from veratryl alcohol oxidation using a UV spectrophotometer at 310 nm. One unit of activity is defined as one micromole of veratryl alcohol oxidized in one min, while the activities are reported in units/L (U/L). The 1,2-bis(3,4-dimethoxyphenyl) propane-1,3-diol is a substrate of this enzyme, whereas 3,4-dimethoxybenzaldehyde, 1-(3,4-dimethoxyphenyl) ethane-1,2-diol, and H_2_O, are its products, as is displayed in Figure 10.

The other method is the Azure B assay. In this method, the relevant reaction assay contains Azure B dye, H_2_O_2_, and sodium tartrate buffer (pH 4.5). The activity is measured at a 615 nm wavelength [256]. This method has been identified as a good assay to reduce the turbidity caused by organic materials under the UV range. Mushrooms have been found as the first LiP producers, namely *T*. *versicolor*, *P*. *ostreatus*, *G*. *lucidum*, and *Bjerkandera* spices [232,257].

#### 4.3.3. Manganese Peroxidase

**Figure 11:**
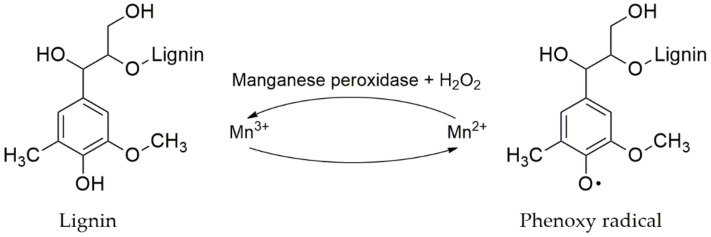
Lignin depolymerisation with manganese peroxidase [259].

Manganese peroxidase (MnP) belongs to the family of oxidoreductases and cannot react directly with the lignin structure [250]. There are two groups: (1) Manganese dependent peroxidase is an extracellular enzyme that requires both H_2_O_2_ for lignin oxidation, Mn^2+^ as a co-factor and (2) Manganese independent peroxidase is an extracellular enzyme that requires H_2_O_2_ in lignin oxidation but does not need Mn^2+^ (Figure 11) [258]. The major substrates of manganese peroxidase are low molecular weight substances and organic acid compounds. In the mechanism cycle of lignin degradation, Mn^2+^ is an electron donor and MnP is oxidized by H_2_O_2_ as follows:

“MnP + H_2_O_2_ → MnP compound I + H_2_O” (1)

“MnP compound I + Mn^2+^ → MnP compound II + Mn^3+^” (2)

“MnP compound II + Mn^2+^ → MnP + Mn^3+^ + H_2_O” (3)

The electron-lacking MnP is nonstable and accepts an electron from Mn^2+^ to Mn^3+^ that then reacts with certain organic acid chelators such as oxalate, malonate, and lactate. The chelated-Mn^3+^ will act as a mediator to oxidize simple phenols, amines, and phenolic lignins. The enzyme can oxidize both phenolic and non-phenolic lignins [260]. The 3,3’-diaminobenzidine (DAB) assay [261] and manganese peroxidase (MnP) assay [262] are the methods used for identification of peroxidase using 0.01% phenol red or 2 mM 2,6-dimethoxyphenol (DMP) as a substrate.

Many mushroom species have been identified as MnP-producing fungi, especially *P. ostreatus* and *Ph. chrysosporium* [263]. Manganese dependent peroxidase is produced from *P. pulmonarius*, which can oxidize both non-phenolic and phenolic compounds for xenobiotic compound degradation. Kuhar and co-workers [264] have reported that MnCl_2_ can induce MnP activity and has a high specificity for Mn^2+^ binding sites.

#### 4.3.4. Versatile Peroxidase

**Figure 12:**
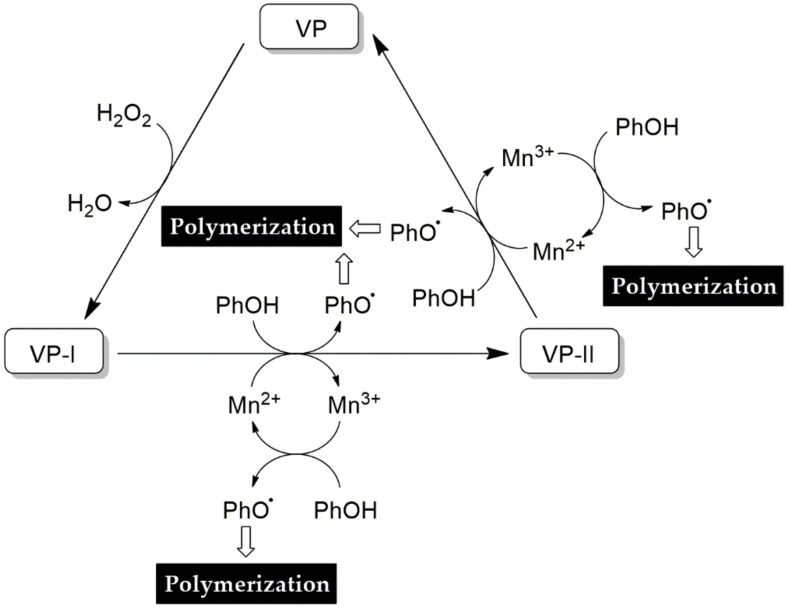
Scheme of the versatile peroxidase catalytic cycle [265].

Versatile peroxidase (VP) is also known as a hybrid peroxidase or polyvalent peroxidase for Mn^2+^ oxidation. VP includes both LiP and MnP activities. Consequently, VP is able to degrade a wider range of substrates than non-hybrid enzymes. VP requires H_2_O_2_ as an electron acceptor to catalyze the oxidative reaction at the heme center with the release of a water molecule [250]. VP is a heme-containing glycoprotein that has a two-channel structure: the wider channel for access to H_2_O_2_ and the narrow channel for access to manganese. Low molecular substrates will be oxidized at the heme center by H_2_O_2_-ferric state binding (heme forming iron peroxide complex). This activated heme complex is able to oxidize the aromatic substrate using Mn^2+^, and then secretes Mn^3+^ and water [265] (Figure 12). VP has been produced by SSF of *P. eryngii* and *P. ostreatus* on wheat straw, sawdust, and banana peels [223,266]. *Pleurotus ostreatus* and *Bjerkandera* sp. were cultured in glucose-peptone broth and glucose ammonium medium using submerged fermentation for VP production [267]. The molecular weight and pI of VP obtained from *P. eryngii* were approximately 40 kDa and 4.1, respectively [268]. The VP activity can be determined by monitoring manganese oxidation and Reactive clack (RB5) decolorization [267].

#### 4.3.5. Dye Decolorizing Peroxidases

Dye decolorizing peroxidases (DyPs) are a new family of glycoproteins that have one heme as a cofactor occurring in basidiomycetous fungi and eubacteria. DyPs require H_2_O_2_ as an electron acceptor and are similar to VP; however, DyPs can oxidize the high-redox potential anthraquinone dyes in addition to typical peroxidase substrates such as RBs, phenols, veratryl alcohol [269,270]. There are four types of DyPs from A to D based on their primary sequences [271]. However, type A DyPs has been reported as the potential type that is most effective in lignin depolymerization. The important characteristic of DyPs is the degradation of hydroxyl-free anthraquinone, which is not a substrate of other peroxidases [270]. DyPs can oxidize certain phenolic compounds such as 2,6-dimethoxyphenol and guaiacol. Only a few types of fungi can produce DyPs, especially type d-DyP, whereas they are mostly present in bacteria (types A, B, and C). The first DyP was discovered in *B. adusta* [272]. The wood-rotting fungi *A. auricula-jadae*, *Mycetinis scorodonius*, *Exidia glandulosa*, *P. sapidus* DSM8266 and *Mycena epipterygia* have also been reported as DyPs producers [273,274]. White-rot fungus, *Irpex lacteus* CD2, exhibited DyPs activity when it was grown in Kirk’s medium containing lignins [275]. Many previous publications have reported that DyPs might be important for the ligninolytic system in white-rot fungi despite the fact that the biological roles of DyPs are unknown in terms of different substrate specificities. The mechanism of DyPs is similar to that of plant peroxidase, which is known to generate transient intermediates (compound I and compound II). The reaction of compound I with 1 eq electrons from a reducing substrate generates the [FeIV = O]^+^ intermediate compound II [271]. The optimum pH value of DyPs is acidic [276]. DyPs activity was assayed by the decolorization of an anthraquinone dye RB19 at 595 nm [275].

### 4.4. Application of Lignocellulolytic Enzymes in Bioprocessing

Enzyme technology possesses great potential to reduce environmental pollution and offers potential benefits in the comprehensive utilization of lignocellulosic biomass. Lignocellulolytic enzymes have received attention because of their potential applications in various agro-industrial bioprocesses, such as the conversion of hemicellulosic biomass to fuels and chemical production, the clarification of juices, the green processing of certain foods and beverages, the enhancement of animal digestibility in feedstock, the delignification of paper and pulp, the improvement of fabric properties in the textile industry and waste utilization [277,278,279]. Cellulase is widely used in the textile and laundry detergent industries as it can play a part in the hydrolysis of cellulose and improve fabric properties for the textile industry and for cleaning textiles in the laundry detergent industry [154,280]. The food and beverage processing industries have used cellulase for the hydrolysis of cellulose during the drying of coffee beans and for the extraction of fruits and vegetables in juice production [281,282]. Cellulase, α-l-arabinofuranosidases and other glycosidases have also been used in brewery and wine production [213,277]. The enzymatic hydrolysis of grapes utilizes α-l-arabinofuranosidases and other glycosidases to enhance the flavor of wine by the release of free terpenols, an important aspect in the development of the aroma in wine. The enzyme treatment by α-l-arabinofuranosidases during sourdough preparation in the bread industry delays the staling process of bread and increases the shelf life of bread [213]. This results in economic benefits in terms of the preservation of bread and bread storage issues. Enzyme technology has a significant potential to improve the properties of pulp. Cellulases, xylanase and other hemicellulases are commonly used enzymes to assist in pulp bleaching for the reduction of environmental pollution loads [283]. Cellulases are used to improve the performance of dissolved pulp [277]. Additionally, α-l-arabinofuranosidases enhance the delignification of pulp in the bleaching process as it can cleave the arabionose side chain that inhibits the action of xylanase [213]. Laccase can be used for lignin removal in prehydrolysis of lignocellulosic biomass [284]. Xylanolytic enzymes have potential applications across food and feed industries [278]. A combination of α-l-arabinofuranosidases with cellulases, pectinases and xylanases enhance the feed digestibility and utilization of polysaccharides in feedstuffs [186,213]. Arabinoxylans are the major non-starch polysaccharide fractions in wheat, which increase digesta viscosity, reduce the digestibility of nutrients and decrease the feed efficiency and growth performance when fed to poultry, especially in broiler chickens [278]. Various reports have revealed the positive effects of MOS on intestinal microflora, along with efficient intestinal structure and function. MOS-based nutrition supplements are widely used in nutrition as a natural additive [279]. The treatment of copra meal rich in β-mannan with mannanase has been reported to reduce the population of *Salmonella* and *Escherichia coli*, increase the level of metabolizable energy and improve the nutrient digestibility in broilers [285]. Olaniyi et al. [207] reported that the treatment of cassava peels and corn cobs with mannanase increased the degradation of the complex carbohydrate fractions in the samples and resulted in increasing the amount of crude protein and certain mineral contents. Kim et al. [273] reported that the supplementation of β-mannanase for diet feeds does not mitigate the heat stress of aged laying hens raised under hot climatic conditions. Saeed et al. [206] describes the promising beneficial effects of β-mannanase in the poultry feed industry as the supplementation of β-mannanase in poultry diets that positively improved blood glucose and anabolic hormone homeostasis, digestible energy, and digestible amino acids. These enzymes have been used as food additives in the poultry raising industry and have been employed in the improvement of nutritional properties of agricultural silage and grain feed.

Manganese peroxidase is an important enzyme associated with the lignin and organic pollutant degradation systems, for instance bioremediation, dye decolorization, pulp bleaching, biomechanical pulping and in the production of a range of highly valuable products that have been obtained from residual lignins [286]. DyPs can be applied in the treatment of wastewater that contain synthetic dyes which are used in the manufacture of textiles, cosmetics, food, and pharmaceuticals. In the food industry, DyPs obtained from *M. scorodonius*, namely the MaxiBright® brand, are used to whiten whey in cheese making [274]. Enzymes have been extensively used in various industries as well as in a lot of the resulting products. Thus, genetic engineering is a powerful tool for the enhancement of ligninolytic enzyme production. White-rot fungus, *Ph. chrysosporium*, is a good model for the study of lignin degradation using DNA technology. The genome sequence encoded several genes such as ten lignin peroxidases, five manganese peroxidases, and several other lignocellulolytic enzymes [287,288]. Laser mutagenesis of *Phellinus igniarius* SJZ2 (mutant) overexpressed Lac activity during 4 h of fermentation and was increased by 36.84% in comparison with the wild type [242]. In addition to the overexpression of Lac in *Saccharomyces cerevisiae* using the laccase III (cvl3) gene obtained from *T. versicolor*, IFO1030 was secreted in the culture (45 U/L) [289]. Lignocellulosic enzymes are obtained from mushrooms, especially white-rot basidiomycetes, which are interesting tools in the biotechnological process that is used in a wide range of lignin substrates.

## 5. Conclusions

The utilization of agro-industrial waste in mushroom cultivation and the production of lignocellulolytic enzymes can facilitate the reduction of some global waste management problems. The cultivation of edible mushrooms using agro-industrial waste represents the bioconversion of that waste into edible protein. Different types of agro-industrial waste can be used for the cultivation of substrates for mushroom cultivation. However, the composition and availability of agro-industrial waste in each area has been considered for the support of mushroom cultivation. Different mushroom species and C/N ratios in substrates are the crucial factors that affect the production and chemical composition of mushrooms. The nitrogen content of agro-industrial waste is low; therefore, this waste is generally associated with other nitrogen sources. The selected suitable substrate and mushroom species are important in obtaining the maximum yields.

Mushrooms seem to be the most important players in lignocellulose degradation by producing both hydrolytic and oxidative enzymes. Hydrolytic enzymes (cellulases and hemicellulases) are known to be responsible for polysaccharide degradation, while oxidative enzymes (ligninases) are responsible for lignin modification and degradation. Current results indicate that agro-industrial waste has been evaluated for its potential use in lignocellulosic enzyme production by mushrooms. However, the variability of waste composition and mushroom species are influential in enzyme production. Therefore, further studies are needed to demine the suitable conditions (substrates, mushroom species and fermentation process) for effective lignocellulosic enzyme production in the pilot study and on the industrial scale.

## Figures and Tables

**Table 1:** Main composition and carbon/nitrogen (C/N) ratio of some agro-industrial wastes.

Agro-Industrial Wastes	Composition (% Dry Weight Basis)			C/N Ratio	Reference
	Cellulose	Hemicellulose	Lignin		
Apple pomace	43	24	20	48/1	[19]
Banana straw	53	29	15	40/1	[20]
Banana leaves	55	20	25	38/1	[21]
Barley straw	23–33	21–22	14–19	82–120/1	[22,23]
Canola straw	22	17	18	33–45/1	[23]
Coconut husk	24–43	3–12	25–45	75–186/1	[24,25]
Coffee husk	43	7	9	40/1	[26]
Corn bran	34	39	49	ND	[25]
Corn cob	35–45	35–44	11–15	50–123/1	[27,28]
Corn stalk	34–61	19–24	7–9	57–80/1	[25,29]
Corn straw	30	25	8	50/1	[25]
Cotton stalk	58	14	22	70–78/1	[22]
Grasses	25–41	25–50	7–30	16–42/1	[30]
Hardwoods	40–55	24–40	18–25	150–450/1	[30]
Oat bran	49	25	18	12/1	[25]
Oat straw	25–40	21–27	17–18	48–83/1	[22,23]
Rice bran	35	25	17	12–48/1	[25]
Rice husk	35	25	20	30–80/1	[31]
Rice straw	32–39	23–24	18–36	35–72/1	[29,32]
Rye straw	38	31	19	82/1	[22]
Beech sawdust	41	33	22	100–331/1	[33]
Birch sawdust	40	36	20	700/1	[33]
Oak sawdust	25–38	18–29	18–25	162–200/1	[31,33]
Pine sawdust	42	25	28	724–1070/1	[33]
Poplar sawdust	44	32	21	46–71/1	[33]
Rubber tree sawdust	38	25	15	177/1	[34]
Spruce sawdust	42	26	28	763–1000/1	[33]
Softwood	45–50	25–35	25–35	310–520/1	[30]
Sorghum stalk	17	25	11	45/1	[25]
Sorghum straw	36	26	8	20–46/1	[35,36]
Pineapple leaf	36	23	27	49/1	[37]
Pineapple peel	22	75	3	77/1	[38]
Potato peel	35	5	4	25/1	[39]
Orange peel	9–14	6–11	1–2	102/1	[40,41]
Lemon peel	12	5	2	ND	[41]
Tomato pomace	9	5	5	ND	[42]
Banana peel	12	10	3	18–29/1	[22]
Soya stalk	35	25	20	20–40/1	[43]
Sugarcane bagasse	30–45	26–36	11–23	50/1	[22,29,44]
Sugarcane straw	36–41	21–31	16–26	70–120/1	[45,46]
Sunflower stalk	42	30	13	97/1	[43]
Oil palm empty fruit bunch	45–51	28–29	12–15	77/1	[47,48]
Water hyacinth	21	34	7	11/1	[10]
Wheat bran	30	50	15	19/1	[25]
Wheat straw	27–38	21–29	18–21	50–80/1	[22,25,49]
Walnut shell	36	28	43	175/1	[50]
Almond shell	38	29	30	61/1	[51]
Chestnut shell	21	16	36	8/1	[51]
Pistachio shell	43	25	16	43/1	[51]
Hazelnut shell	55	34	35	50–58/1	[52]
Olive oil cake	31	21	26	14–17/1	[53]
Oil palm cake	64	15	5	ND	[54]
Sunflower oil cake	25	12	8	ND	[54]
Cotton seed hull	31	20	18	59–67/1	[55]

“ND” = not determined.

**Table 2:** The carbon/nitrogen ratio in substrate to obtain the highest yield of some mushroom species.

Mushroom Species	C/N Ratio (%)			Reference
	Minimum	Optimum	Maximum	
*Agaricus bisporus*	16/1	19/1	22/1	[68]
*Agaricus bitorquis*	16/1	19/1	22/1	[69]
*Agaricus brasiliensis*	10/1	26–28/1	50/1	[70]
*Agaricus brunescens*	16/1	19/1	21/1	[71]
*Agaricus subrufescens*	16/1	27/1	33/1	[72]
*Lentinula edodes*	25/1	30–35/1	55/1	[73]
*Lentinus sajor-caju*	40/1	45–55/1	90/1	[74]
*Pleurotus cornucopiae*	40/1	45–55/1	97/1	[75]
*Pleurotus eryngii*	40/1	45–55/1	70/1	[75]
*Pleurotus flabellatus*	40/1	45–60/1	100/1	[76]
*Pleurotus florida*	40/1	45–60/1	150/1	[77,78]
*Pleurotus ostreatus*	40/1	45–60/1	90/1	[78]
*Flammulina velutipes*	ND	30/1	ND	[79]
*Ganoderma lucidum*	ND	70–80/1	ND	[80]
*Volvariella volvacea*	ND	40–60/1	ND	[81]

“ND” = not determined.

**Table 3:** Biological efficiency and chemical composition of some mushrooms grown on the non-combination of agro-industrial wastes.

Agro-Industrial Wastes	Mushroom Species	Biological Efficacy (%)	Chemical Composition (% Dry Weight)					Reference
			Crude Protein	Carbohydrate	Fat	Fiber	Ash	
Wheat straw	*Agaricus bisporus*	47.2–51.1	21.0–27.0	38.0–48.0	3.0–4.0	17.0–23.3	8.0–11.0	[83,84]
	*Agaricus subrufescens*	53.7	28.4	63.2	1.6	6.2	6.8	[85]
	*Agrocybe cylindracea*	61.4	1.5	89.6	0.3	40.4	8.6	[86]
	*Hericium erinaceus*	39.4–43.5	26.8	58.9	3.7	ND	10.5	[87]
	*Lentinula edodes*	66.0–93.1	15.2–15.4	63.7–65.7	1.1–1.5	ND	3.8–4.4	[88]
	*Lentinus sajor-caju*	74.9	22.9	56.0	2.6	7.1	6.6	[89]
	*Pleurotus citrinopileatus*	98.3–105.6	25.3	64.0	2.7	ND	8.1	[90]
	*Pleurotus columbinus*	69.2	2.9	25.9	0.42	5.4	8.5	[91]
	*Pleurotus eous*	75.1	19.5	50.2	2.6	7.8	6.0	[92]
	*Pleurotus eryngii*	48.2	21.5	56.0	2.4	13.5	7.6	[93]
	*Pleurotus florida*	66.4	27.9	51.2	2.4	12.2	8.7	[94]
	*Pleurotus ostreatus*	22.6–52.6	11.6–14.6	47.5–74.4	1.8–2.5	19.1–27.1	8.6–12.0	[86,95]
	*Pleurotus* *sapidus*	62.2	14.9	48.5	2.0	7.3	6.2	[96]
Barley straw	*Lentinula edodes*	64.1–88.6	15.1–16.8	75.1–77.7	1.9–2.2	ND	5.2–5.8	[88]
	*Pleurotus ostreatus*	21.3	12.8	54.7	29.9	0.9	1.2	[95]
Oat straw	*Agaricus bisporus*	47.2–52.9	26.8–36.2	ND	2.3–3.1	6.6–10.3	9.8–11.3	[97]
	*Ganoderma lucidum*	2.3	9.9	ND	ND	ND	1.0	[98]
Rice straw	*Lentinula edodes*	48.7	16.2	78.0	6.0	1.5	3.4	[99]
	*Lentinus sajor-caju*	78.3	23.4	55.0	2.4	7.9	6.8	[89]
	*Hericium erinaceus*	33.9	24.1	60.5	4.2	ND	11.3	[87]
	*Pleurotus citrinopileatus*	76.5–89.2	22.8	64.9	3.2	ND	91	[90]
	*Pleurotus columbinus*	71.4	4.8	27.3	0.3	5.0	7.7	[91]
	*Pleurotus eous*	79.8	29.3	48.0	2.4	8.0	6.2	[92]
	*Pleurotus eryngii*	45.9	21.8	53.0	1.9	13.8	8.7	[93]
	*Pleurotus pulmonarius*	23.5	21.1	ND	5.2	7.0	6.9	[100]
	*Pleurotus ostreatus*	25.6–84.6	12.5–23.4	55.3–57.4	2.8–16.2	7.7–0.7	6.3–13.6	[95,101]
	*Pleurotus* *sapidus*	64.7	23.4	45.6	1.6	8.0	6.4	[96]
	*Pleurotus djamor*	82.7	24.8	37.7	3.1	22.0	8.3	[102]
	*Volvariella volvacea*	10.2–15.0	36.9–38.1	42.8–42.3	0.8–1.0	4.4–6.0	9.0–10.3	[103,104,105]
	*Corprinus comatus*	18.0	10.9	76.6	1.9	ND	20.5	[106]
Corn straw	*Pleurotus florida*	31.6	26.3	31.3	0.5	19.6	5.2	[107]
	*Volvariella volvacea*	ND	23.0	13.9	1.4	36.6	11.9	[108]
Corn cob	*Agrocybe cylindracea*	33.5	14.8	72.4	2.9	17.0	10.1	[86]
	*Pleurotus columbinus*	79.1	1.9	28.5	0.2	4.12	9.3	[91]
	*Pleurotus cystidiosus*	50.1	24.5	40.6	3.0	24.3	7.57	[109]
	*Pleurotus eryngii*	51.8	23.8	54.8	1.9	9.7	7.0	[93]
	*Pleurotus florida*	55.0	29.1	38.2	0.9	22.8	3.5	[107]
	*Pleurotus ostreatus*	31.7–66.1	15.4–29.7	30.8–73.4	2.7–3.4	13.8–29.8	7.1—8.0	[86,109]
Banana leaves	*Pleurotus ostreatus*	ND	15.0	24.9	2.2	5.1	11.2	[62,110]
	*Pleurotus pulmonarius*	17.9	16.9–23.5	26.2	1.9–5.5	5.8–7.2	6.4–10.3	[62,100]
	*Volvariella volvacea*	15.2	23.9	ND	ND	8.1	6.1	[111]
Soya stalk	*Lentinus sajor-caju*	83.0	25.8	52.2	2.8	6.7	7.3	[89]
	*Pleurotus eous*	82.3	30.5	50.5	2.6	9.0	6.5	[92]
	*Pleurotus ostreatus*	85.2	24.7	53.2	2.8	7.2	6.7	[101]
	*Pleurotus columbinus*	90.6	7.4	33.3	0.4	5.1	9.2	[91]
	*Pleurotus florida*	87.6	23.5	57.8	2.5	8.0	8.0	[112]
	*Pleurotus sapidus*	72.7	26.8	24.9	2.1	7.5	7.0	[96]
Sunflower stalk	*Lentinus sajor-caju*	63.1	21.0	50.7	2.8	7.7	6.9	[89]
	*Pleurotus eous*	61.5	27.4	52.0	2.2	7.9	5.2	[92]
	*Pleurotus sapidus*	45.9	20.1	48.5	2.4	7.3	6.2	[96]
Oil palm empty fruit bunch	*Schizopyllum commune*	3.7	6.1	37.4	4.5	0.01	1.94	[113]
	*Volvariella volvacea*	3.6–6.5	33.5–41.0	27.9–45.7	3.7–5.1	7.7–16.0	9.4–9.9	[114]
Cotton stalk	*Pleurotus florida*	25.1	29.8	37.3	2.2	19.4	8.7	[115]
	*Pleurotus pulmonarius*	42.3	29.3	44.5	3.1	11.3	9.2	[115]
	*Pleurotus ostreatus*	44.3	30.1	40.2	2.1	17.2	8.4	[115]
Rice husk	*Pleurotus ostreatus*	9.5	5.9	48.5	30.9	0.3	14.3	[95]
Sugarcane bagasse	*Lentinula edodes*	130.0–133.0	13.1–13.8	73.0–78.9	0.9–1.0	ND	6.2–7.1	[116]
	*Pleurotus cystidiosus*	49.5	22.1	45.2	2.3	22.8	7.5	[109]
	*Pleurotus djmor*	101.7	25.1	45.2	2.1	9.1	4.1	[117]
	*Pleurotus eryngii*	41.3	20.5	49.0	3.1	8.0	7.8	[93]
	*Pleurotus florida*	75.6	8.7	ND	4.0	2.5	0.3	[118]
	*Pleurotus ostreatus*	65.7	27.1	34.9	2.0	29.3	6.7	[109]
Sugarcane straw	*Lentinula edodes*	83.0–98.0	14.4	72.5–78.2	0.7–0.9	NR	6.4–6.5	[116]
Cottonseed hull	*Pleurotus florida*	13.6	20.0	61.2	11.9	11.9	5.5	[119]
	*Pleurotus ostreatus*	8.9	17.5	65.9	1.2	10.2	5.2	[119]
Cassava peel	*Pleurotus ostreatus*	24.0–26.1	10.5–10.7	73.0–74.6	2.1–2.2	8.5–8.9	7.5–7.7	[120]
	*Volvariella volvacea*	0.6-2.3	11.5–14.3	51.4–53.4	2.4–2.6	0.4–0.5	5.0–6.2	[121]
Hardwood sawdust	*Hericium erinaceus*	47.5–50.3	24.8	60.9	3.6	ND	10.6	[87]
Acacia sawdust	*Pleurotus cystidiosus*	36.3	15.7	55.9	2.1	20.1	6.3	[109]
	*Pleurotus ostreatus*	46.4	19.5	51.3	1.3	22.0	5.9	[109]
Beech sawdust	*Agrocybe cylindracea*	38.3	18.4	70.3	3.4	15.0	8.2	[86]
	*Ganoderma lucidum*	61.2	16.8	77.9	2.2	47.9	3.1	[122]
	*Pleurotus ostreatus*	46.8	16.1	73.6	3.5	15.8	6.2	[86]
Sawdust	*Auricularia polytricha*	13.9–44.6	10.2	78.4	0.9	ND	4.2	[123]
	*Pleurotus columbinus*	89.1	1.7	25.0	0.2	4.6	9.1	[91]
	*Pleurotus citrinopileatus*	38.4–51.6	24.1	65.6	2.6	ND	7.8	[90]
	*Pleurotus eryngii*	35.5	19.5	52.5	2.4	7.8	7.5	[93]

“ND” = not determined.

**Table 4:** Biological efficiency and chemical composition of some mushrooms grown on the combination of agro-industrial wastes.

Agro-Industrial Wastes	Mushroom Species	Biological Efficacy (%)	Chemical Composition (% Dry Weight)					Reference
			Crude Protein	Carbohydrate	Fat	Fiber	Ash	
Soya stalk (50%) + rice straw (50%)	*Pleurotus florida*	85.2	22.7	54.9	2.6	7.6	6.5	[111]
	*Pleurotus ostreatus*	81.7	23.0	50.5	2.7	7.7	6.4	[124]
Soya stalk (50%) + wheat straw (50%)	*Pleurotus florida*	78.2	22.4	57.1	2.3	7.5	6.4	[111]
	*Pleurotus ostreatus*	77.7	21.1	52.0	2.6	7.4	6.2	[124]
Wheat straw (50%) + Rice straw (50%)	*Hericium erinaceus*	32.5–37.2	25.6	60.6	3.9	ND	9.7	[87]
	*Pleurotus florida*	72.3	20.2	53.9	2.3	7.4	6.5	[111]
	*Pleurotus ostreatus*	71.8	20.3	56.0	2.6	7.5	5.9	[124]
Oat straw (80%) + wheat bran (20%)	*Ganoderma lucidum*	2.0–2.5	10.6–12.5	ND	ND	47.8–57.7	1.3–1.5	[98]
Cotton stalk (50%) + Cottonseed hull (50%)	*Pleurotus florida*	17.3	24.5	52.0	3.2	13.2	7.1	[119]
	*Pleurotus ostreatus*	20.2	22.8	58.0	2.9	10.8	5.5	[119]
Acacia sawdust (50%) + corn cob (50%)	*Pleurotus cystidiosus*	43.6	21.4	44.8	2.8	23.6	7.3	[109]
	*Pleurotus ostreatus*	58.8	18.7	46.9	3.3	24.5	6.7	[109]
Acacia sawdust (50%) + sugarcane bagasse (50%)	*Pleurotus cystidiosus*	41.1	25.6	37.5	1.8	28.5	6.8	[109]
	*Pleurotus ostreatus*	58.9	24.2	37.8	2.5	28.8	6.7	[109]
Sugarcane bagasse (50%) + grasses (50%)	*Agaricus brasiliensis*	44.3	28.3	ND	1.6	5.8	6.7	[125]
Rubber tree sawdust (50%) + rice straw (50%)	*Flammulina velutipes*	123.9	17.0–27.0	58.0–87.0	1.8–7.3	ND	7.3–10.4	[126]
Beech sawdust (50%) + olive pruning residues (50%)	*Ganoderma lucidum*	20.5	15.3	79.3	2.0	43.8	3.4	[122]
Wheat straw (50%) + olive pruning residues (50%)	*Pleurotus ostreatus*	56.8	19.9	71.7	1.9	16.5	6.5	[122]
Sawdust (90%) + rice bran (10%)	*Pleurotus eous*	48.4–68.1	27.8	28.6	5.6	17.3	4.9	[127]
Sugarcane bagasse (50%) + rice straw (50%)	*Lentinus sajor-caju*	83.9	30.9	33.8	ND	24.5	6.9	[128]
Cassava peel (50%) + corn cobs (50%)	*Pleurotus ostreatus*	31.1–33.7	10.6–10.8	73.6–74.8	2.1–2.2	8.6–8.9	7.3–7.8	[120]
Hard wood sawdust (50%) + rice straw (50%)	*Hericium erinaceus*	36.5–44.2	25.1	59.8	4.0	ND	11.0	[87]
Hard wood sawdust (50%) + wheat straw (50%)	*Hericium erinaceus*	41.4–46.5	24.7	60.8	4.2	ND	10.3	[87]
Hardwood sawdust (30%) + corn stalk (60%) + rice bran (10%)	*Auricularia polytricha*	27.3–41.0	11.1	76.1	0.9	ND	4.8	[129]

“ND” = not determined.

**Table 5:** Production of enzymes in solid state fermentation of cellulose degradation by some mushrooms using agro-industrial wastes.

Enzyme	Agro-Industrial Wastes	Mushroom Species	Activity	Reference
Total cellulase	Wheat straw	*Lentinula edodes*	45–60 U/mL	[151]
		*Pleurotus dryinus*	41–120 U/mL	[151,160]
		*Pleurotus ostreatus*	665–1185 U/mL	[151]
		*Pleurotus tuber-regium*	505 U/mL	[151]
		*Fomitopsis* sp.	3.5 U/gds	[159]
	Tree leaves (*Fagus sylvatica*)	*Lentinula edodes*	40–45 U/mL	[151]
		*Pleurotus dryinus*	205 U/mL	[151]
		*Pleurotus ostreatus*	14–15 U/mL	[151]
		*Pleurotus tuber-regium*	20 U/mL	[151]
	Sorghum straw	*Pycnosporus sanguineus*	0.8 U/gds	[153]
		*Pleurotus ostreatus*	1.3 U/gds	[153]
		*Pleurotus eryngii*	0.7 U/gds	[153]
		*Phanerochaete chrysosporium*	1.1 U/gds	[153]
		*Trametes versicolor*	1.0 U/gds	[153]
	Eucalyptus wood chip	*Wolfiporia cocos*	1.4–8.3 U/gds	[161]
		*Laetiporeus sulfureus*	0.8–15.4 U/gds	[161]
		*Poria medulla-panis*	0.4–3.4 U/gds	[161]
		*Pycnoporus coccineus*	1.8–3.7 U/gds	[161]
		*Phlebia tremellosa*	0.6–2.0 U/gds	[161]
		*Trametes versicolor*	0.8–4.0 U/gds	[161]
Endoglucanase	Wheat straw	*Lentinus tigrinus*	1230 U/gds	[156]
		*Lentinula edodes*	180–345 U/mL	[151]
		*Pleurotus dryinus*	910 U/mL	[151]
		*Pleurotus ostreatus*	185-245 U/mL	[151]
		*Pleurotus tuber-regium*	150 U/mL	[151]
		*Piptoporus betulinus*	83.5 U/gds	[157]
	Tree leaves (*Fagus sylvatica*)	*Lentinula edodes*	40–65 U/mL	[151]
		*Pleurotus dryinus*	1130 U/mL	[151]
		*Pleurotus ostreatus*	25–1300 U/mL	[151]
		*Pleurotus tuber-regium*	150 U/mL	[151]
	Sorghum straw	*Pycnosporus sanguineus*	2.0 U/gds	[153]
		*Pleurotus ostreatus*	2.3 U/gds	[153]
		*Pleurotus eryngii*	1.4 U/gds	[153]
		*Phanerochaete chrysosporium*	4.0 U/gds	[153]
		*Trametes versicolor*	2.2 U/gds	[153]
	Sugarcane bagasse	*Lentinus sajor-caju*	13.9–18.9 U/gds	[162]
		*Pleurotus ostreatus*	3.0 U/gds	[163]
	Sawdust	*Trametes trogii*	504 U/gds	[164]
		*Coriolus versicolor*	0.6 U/gds	[165]
		*Ganoderma applanatum*	0.1 U/gds	[165]
		*Pycnoporus sanguineus*	0.6 U/gds	[165]
		*Trametes villosa*	0.2 U/gds	[165]
		*Pleurotus ostreatus*	3.0 U/gds	[163]
		*Lentinus sajor-caju*	0.9 U/gds	[163]
	Rice straw	*Pleurotus ostreatus*	7.1 U/gds	[163]
		*Lentinus sajor-caju*	1.9 U/gds	[163]
	Oak sawdust	*Grifola frondosa*	12.3 U/gds	[152]
	Pine chip	*Coriolus versicolor*	2.4 U/gds	[165]
		*Ganoderma applanatum*	2.8 U/gds	[165]
		*Pycnoporus sanguineus*	4.8 U/gds	[165]
		*Trametes villosa*	3.9 U/gds	[165]
	Green tea waste	*Microporus xanthopus*	38.6 U/gds	[166]
	Wheat straw	*Fomitopsis* sp.	53.6 U/gds	[159]
Exoglucanase	Oak sawdust	*Grifola frondosa*	16.2 U/gds	[152]
	Rice straw	*Pleurotus ostreatus*	2.0 U/gds	[163]
		*Lentinus sajor-caju*	1.8 U/gds	[163]
	Sugarcane bagasse	*Pleurotus ostreatus*	7.0 U/gds	[163]
		*Lentinus sajor-caju*	2.0 U/gds	[163]
	Sawdust	*Pleurotus ostreatus*	2.8 U/gds	[163]
		*Lentinus sajor-caju*	0.6 U/gds	[163]
	Corn stover	*Irpex lacteus*	69.3 U/gds	[167]
β -Glucosidase	Sorghum straw	*Pycnosporus sanguineus*	0.4 U/gds	[153]
		*Pleurotus ostreatus*	0.2 U/gds	[153]
		*Pleurotus eryngii*	0.2 U/gds	[153]
		*Phanerochaete chrysosporium*	1.1 U/gds	[153]
		*Trametes versicolor*	1.9 U/gds	[153]
	Eucalyptus wood chip	*Wolfiporia cocos*	8.3–42.0 U/gds	[161]
		*Laetiporeus sulfureus*	7.6–37 U/gds	[161]
		*Poria medulla-panis*	2.7–10.5 U/gds	[161]
		*Pycnoporus coccineus*	8.0–22.0 U/gds	[161]
		*Phlebia tremellosa*	3.8–15.6 U/gds	[161]
		*Trametes versicolor*	3.8–20.0 U/gds	[161]
	Oak sawdust	*Grifola frondosa*	2.3 U/gds	[152]
	Rice straw	*Pleurotus ostreatus*	2.5 U/gds	[163]
		*Lentinus sajor-caju*	1.2 U/gds	[163]
	Sugarcane bagasse	*Pleurotus ostreatus*	3.5 U/gds	[163]
		*Lentinus sajor-caju*	2.6–12.3 U/gds	[162,163]
	Sawdust	*Pleurotus ostreatus*	2.2 U/gds	[163]
		*Lentinus sajor-caju*	0.2 U/gds	[163]
		*Coriolus versicolor*	0.5 U/gds	[165]
		*Ganoderma applanatum*	0.4 U/gds	[165]
		*Pycnoporus sanguineus*	0.4 U/gds	[165]
		*Trametes villosa*	0.5 U/gds	[165]
		*Trametes trogii*	0.89 U/gds	[164]
	Pine chip	*Coriolus versicolor*	0.3 U/gds	[165]
		*Ganoderma applanatum*	0.1 U/gds	[165]
		*Pycnoporus sanguineus*	0.8 U/gds	[165]
		*Trametes villosa*	0.5 U/gds	[165]
	Wheat straw	*Piptoporus betulinus*	78.8 U/gds	[157]
		*Pleurotus dryinus*	401 U/gds	[160]
		*Lentinula edodes*	0.1 U/gds	[168]
	Sorghum straw	*Pleurotus eryngii*	0.23 U/gds	[153]

**Table 6:** Production of enzymes in solid state fermentation of hemicellulose degradation by some mushrooms using agro-industrial wastes.

Enzyme	Agro-Industrial Wastes	Mushroom Species	Activity	Reference
Total xylanase	Tree leaves (*Fagus sylvatica*)	*Lentinula edodes*	85–200 U/mL	[151]
		*Pleurotus dryinus*	2145 U/mL	[151]
		*Pleurotus ostreatus*	160–1400 U/mL	[151]
		*Pleurotus tuber-regium*	155 U/mL	[151]
	Wheat straw	*Lentinula edodes*	195–275 U/mL	[151]
		*Pleurotus dryinus*	1450 U/mL	[151]
		*Pleurotus ostreatus*	260–735 U/mL	[151]
		*Pleurotus tuber-regium*	260 U/mL	[151]
	Wheat bran	*Fomes fomentarius*	7–63 U/mL	[154]
		*Ganoderma applanatum*	3 U/mL	[154]
		*Pleurotus ostreatus*	16 U/mL	[154]
		*Trametes hirsuta*	8 U/mL	[154]
		*Trametes ochracea*	35 U/mL	[154]
		*Trametes versicolor*	21 U/mL	[154]
		*Trametes pubescens*	32 U/mL	[154]
		*Trametes biforme*	19 U/mL	[154]
Endo-1,4-β-xylanase	Rice straw	*Pleurotus ostreatus*	21 U/gds	[195]
	Sawdust	*Pleurotus ostreatus*	9 U/gds	[195]
	Sugarcane bagasse	*Ganoderma lucidum*	33 U/gds	[196]
	Tomato pomace	*Pleurotus ostreatus*	9 U/gds	[197]
		*Trametes versicolor*	50 U/gds	[197]
	Jerusalem artichoke stalk	*Schizophyllum commune*	106 U/gds	[198]
	Oak leaves	*Marasmius quercophilus*	73 U/gds	[199]
		*Mycena inclinata*	105 U/gds	[199]
		*Pholiota lenta*	83.2 U/gds	[199]
	Wheat straw	*Pleurotus citrinopileatus*	0.12 U/gds	[200]
		*Pleurotus ostreatus*	0.14 U/gds	[200]
	Pine wood chip	*Ceriporiopsis subvermispora*	0.25 U/gds	[201]
	Eucalyptus wood chip	*Ceriporiopsis subvermispora*	0.12 U/gds	[201]
	Soya bran	*Fomes sclerodermeus*	31 U/gds	[202]
	Rice bran mixed rice husk	*Leucoagaricus meleagris*	0.8 U/gds	[203]
	Sugarcane bagasse mixed wheat bran	*Pleurotus ostreatus*	8.7 U/gds	[204]
		*Ganoderma lucidum*	16.3 U/gds	[204]
		*Trametes versicolor*	36.7 U/gds	[204]
1,4-β-Xylosidase	Oak leaves	*Marasmius quercophilus*	1.7 U/gds	[199]
		*Mycena inclinata*	5.8 U/gds	[199]
		*Pholiota lenta*	1.6 U/gds	[199]
	Pine wood chip	*Ceriporiopsis subvermispora*	4.4 U/gds	[201]
	Eucalyptus wood chip	*Ceriporiopsis subvermispora*	2.6 U/gds	[201]
	Wheat straw	*Pleurotus citrinopileatus*	11.5 U/gds	[200]
		*Pleurotus ostreatus*	14.3 U/gds	[200]
	Sugarcane bagasse mixed wheat bran	*Ganoderma lucidum*	0.4 U/gds	[204]
		*Trametes versicolor*	1.5 U/gds	[204]
Endo-1,4-β-mannanase	Oak leaves	*Marasmius quercophilus*	3.4 U/gds	[199]
		*Mycena inclinata*	3.2 U/gds	[199]
		*Pholiota lenta*	11.8 U/gds	[199]
	Pine wood chip	*Ceriporiopsis subvermispora*	90.4 U/gds	[201]
	Eucalyptus wood chip	*Ceriporiopsis subvermispora*	52.2 U/gds	[201]
1,4-β-Mannosidase	Oak leaves	*Marasmius quercophilus*	5.9 U/gds	[199]
		*Mycena inclinata*	4.2 U/gds	[199]

**Table 7:** Production of enzymes in solid state fermentation of lignin degradation by some mushrooms using agro-industrial wastes.

Enzyme	Agro-Industrial Wastes	Mushroom Species	Activity	Reference
Laccase	Tree leaves (*Fagus sylvatica*)	*Lentinula edodes*	7–52 U/L	[151]
		*Pleurotus dryinus*	16 U/L	[151]
		*Pleurotus ostreatus*	6.3–8.0 U/L	[151]
		*Pleurotus tuber-regium*	2.1 U/L	[151]
	Wheat straw	*Lentinula edodes*	3.6–5.2 U/L	[151]
		*Pleurotus dryinus*	5.7 U/L	[151]
		*Pleurotus ostreatus*	1.1–10.1 U/L	[151]
		*Pleurotus tuber-regium*	10 U/L	[151]
		*Pleurotus citrinopileatus*	1.2–3.7 U/gds	[200]
	Wheat bran	*Fomes fomentarius*	7430–17510 U/L	[154]
		*Ganoderma applanatum*	1910 U/L	[154]
		*Pleurotus ostreatus*	9210 U/L	[154]
		*Trametes hirsuta*	7350 U/L	[154]
		*Trametes ochracea*	3930 U/L	[154]
		*Trametes versicolor*	17860 U/L	[154]
		*Trametes pubescens*	5319 U/L	[154]
		*Trametes biforme*	4960 U/L	[154]
	Wheat bran mixed corn straw	*Trametes versicolor*	32.1 U/gds	[227]
Laccase	Corn stalk	*Trametes versicolor*	2,765.81 U/L	[228]
	Sawdust	*Coriolopsis gallica*	200 U/gds	[229]
	Sugarcane bagasse	*Pleurotus ostreatus*	151.6 U/gds	[230]
	Oat husk	*Cerrena unicolor*	28.2 U/gds	[231]
	Pineapple leaves	*Ganoderma lucidum*	42.7 U/gds	[232]
	Rice bran mixed wheat bran	*Stereum ostrea*	24962 U/L	[233]
	Rice straw	*Schizophyllum commune*	431.2 U/gsd	[234]
	Sugarcane bagasse mixed wheat bran	*Ganoderma lucidum*	9.4 U/gds	[204]
		*Pleurotus ostreatus*	2.1 U/gds	[204]
		*Trametes versicolor*	1.9 U/gds	[204]
	Soya bran	*Fomes sclerodermeus*	14.5 U/gds	[202]
Manganese peroxidase	Tree leaves (*Fagus sylvatica*)	*Lentinula edodes*	1.0–6.7 U/L	[151]
		*Pleurotus dryinus*	5.7 U/L	[151]
		*Pleurotus ostreatus*	7–15 U/L	[151]
		*Pleurotus tuber-regium*	20 U/L	[151]
	Wheat straw	*Lentinula edodes*	20–55 U/L	[151]
		*Pleurotus dryinus*	13 U/L	[151]
		*Pleurotus ostreatus*	7–12 U/L	[151]
		*Pleurotus tuber-regium*	2.2 U/L	[151]
		*Pleurotus citrinopileatus*	4.9 U/gds	[200]
	Wheat bran	*Fomes fomentarius*	350 U/L	[154]
		*Pleurotus ostreatus*	20 U/L	[154]
		*Trametes versicolor*	20–50 U/L	[154]
		*Trametes biforme*	570 U/L	[154]
	Oat husk	*Cerrena unicolor*	20.4 U/gds	[231]
	Pineapple leaves	*Ganoderma lucidum*	82.7 U/gds	[232]
	Rice bran mixed wheat bran	*Stereum ostrea*	3895 U/L	[233]
	Rice straw	*Schizophyllum commune*	1964 U/gsd	[234]
	Eucalyptus sawdust	*Lentinula edodes*	700 U/gds	[235]
	Soya bran	*Fomes sclerodermeus*	14.5 U/gds	[202]
	Sugarcane bagasse mixed wheat bran	*Ganoderma lucidum*	1.9 U/gds	[204]
		*Pleurotus ostreatus*	2.3 U/gds	[204]
		*Tramets versicolor*	2.1 U/gds	[204]
	Barley husk	*Bjerkandera adusta*	510 U/kgds	[236]
Lignin peroxidases	Jatropha waste	*Pleurotus ostreatus*	49916 U/L	[237]
	Corn cob	*Ganoderma lucidum*	561.4 U/gds	[238]
	Pineapple leaves	*Ganoderma lucidum*	287.5 U/gds	[232]
	Rice bran mixed wheat bran	*Stereum ostrea*	72.8 U/L	[233]
	Rice straw	*Schizophyllum commune*	1467.3 U/gsd	[234]
	Barley husk	*Bjerkandera adusta*	1700 U/kgds	[236]
	Sugarcane bagasse mixed wheat bran	*Ganoderma lucidum*	0.6 U/gds	[204]
		*Pleurotus ostreatus*	0.5 U/gds	[204]
		*Trametes versicolor*	0.7 U/gds	[204]
Versatile peroxidase	Banana peel	*Pleurotus eryngii*	36 U/gds	[239]
